# The dynamic association between Frailty, CD4 and CD4/CD8 ratio in people aging with HIV

**DOI:** 10.1371/journal.pone.0212283

**Published:** 2019-02-14

**Authors:** Giovanni Guaraldi, Stefano Zona, Ana Rita Silva, Marianna Menozzi, Giovanni Dolci, Jovana Milic, Federica Carli, Cristina Mussini

**Affiliations:** 1 Infectious Diseases Clinic, University of Modena and Reggio Emilia, Modena, Italy; 2 Department of Infectious Diseases, Hospital Beatriz Ângelo, Loures, Portugal; 3 Clinical and Experimental Medicine PhD Program, University of Modena and Reggio Emilia, Modena, Italy; Katholieke Universiteit Leuven Rega Institute for Medical Research, BELGIUM

## Abstract

**Objective:**

To investigate the association between current CD4+ T-cell count and CD4/CD8+ ratio with severity of frailty among people aging with HIV.

**Methods:**

Cross-sectional observational study analysing data from all study visits in the ongoing prospective Modena HIV Metabolic Clinic Cohort between 2006 and 2015. Frailty severity was assessed using a frailty index (FI). We visualized the relationships between frailty index score and current CD4 cell count and CD4/CD8 ratio on two different curves adjusted for age, sex, and duration of HIV infection.

**Results:**

Frailty index scores exhibited an inverse relationship with current CD4 count, up to 900 cells/μL. The CD4/CD8 ratio was inversely correlated with frailty index both below and above the cut-off of 900 CD4 cells/μL.

**Conclusions:**

Frailty in PLWH is inversely associated with both immune-activation, depicted by CD4/CD8 ratio and immune-deficit depicted by CD4 count. The first association shows a linear shape while the second shows a hook-shape with a turning point at 900 cells. Above this cut off level CD4 do not represent a significant risk factor for frailty.

## Introduction

Aging is associated with an increased risk of several adverse health outcomes, including illnesses, disabilities and death. The aging process has great inter-variability, and those at increased risk of adverse outcomes are said to be frail [1,2]. This is also true within groups of people suffering from the same disease, including people living with HIV (PLWH) [3–5].

With the aging of PLWH, we urgently need to better understand how the progression of frailty might be prevented or delayed among this group. Current CD4+ T-cell count is the modifiable covariate most often associated with frailty [3–10]. This relationship has proven tricky to understand, as several recent reports identified no association between CD4 cell count and severity of frailty, especially among study participants with higher current CD4 cell counts [11–14].

This inconsistency may reflect the fact that immune status and aging contribute to frailty in complex ways [15,16]. CD4 cell depletion is associated with a shortened life expectancy, in part due to an increased risk for inflammatory, age-related chronic conditions, including heart disease and cancers [17–19]. Conversely, higher CD4 counts are associated with longer life expectancy and appear to be protective against these diseases [20,21]. This can paradoxically confer risk of other age-related conditions, given that people with higher CD4 counts generally live longer and the strongest risk factor for frailty and for many chronic inflammatory diseases is aging itself [22].

Based on this understanding, our group previously proposed a theoretical U-shaped association between current CD4 count and frailty severity among PLWH [23].

Greater severity of frailty may be associated with lower CD4 counts among some patients, while also being associated with higher CD4 counts among patients who survive to older ages and accumulate traditional risk factors for age-related diseases and frailty, independently of effective antiretroviral therapy [24].

Further complicating this relationship, immune depletion and chronic immune activation each appear to contribute to risk for inflammatory, age-related diseases among PLWH [25]. Both chronic HIV infection and aging lead to T cell activation and progressive accumulation of terminally differentiated T cells (e.g. CD8+ effector cells), a reduction in naïve T cells, a lower CD4/CD8 ratio, and increased levels of multiple inflammatory markers [26]. This leads to a state of immune senescence characterized by low-level chronic inflammation and an impaired ability to mount adequate immune response to challenges [27]. CD4/CD8 ratio is a surrogate marker of immune senescence [28], and is inversely correlated with the risk for inflammatory and/or age-related disease [7,29–32].

In this study, our primary objective was to assess if a large cohort of people aging with HIV demonstrates this U-shaped relationship between current CD4 count and severity of frailty on both down-sloping and up-sloping portions of the U-curve.

Our secondary objective was to assess the relationship between frailty severity and current CD4/CD8 ratio. We hypothesized that CD4/CD8 ratio would be inversely associated with frailty in both portions.

## Methods

Data were collected from participants attending the ongoing prospective Modena HIV Metabolic Clinic (MHMC) Cohort Study [33,34], undergoing effective antiretroviral (ART) treatment (viral load <40 copies/ml), with available frailty and viro-immunological assessments, having accessed the clinic from January 2006 to December 2015.

Exclusion criteria were detectable viral load, both at baseline and in any subsequent visits, i.e. patients were censored if they were found with detectable viral load.

This outpatient clinic is a multidisciplinary tertiary care centre for the management of non-infectious comorbidities in HIV infected patients followed both at the Modena HIV Metabolic Clinic and from HIV care centres throughout Italy.

The association between frailty index and CD4 and CD8 cell count was assessed at each study visit, so that individuals could contribute more than once.

Frailty severity was assessed using a 37-item frailty index, as previously described [3].

A frailty index calculates the proportion of age-related health deficits that a person has accumulated out of a selection of at least 30 health variables, 37 in our setting. Health variables used to calculate frailty index are shown in S1 Table. Any health variable (e.g. signs and symptoms of disease, laboratory measures, and self-reported data) can be included as an item in a frailty index as long as the variables describe potential age-related health problems, and as a group include multiple physiological systems [35]. The frailty index approach is among the most common methods of measuring frailty [36]. Frailty was defined as FI value above a cut off of 0.31 as it was representative of the median of the individuals in the cohort and similar to previous published studies by our cohort.

For the purpose of description, we also depict the proportion of patients with Physical frailty Phenotype (PFP) in a subset of patients in which this data was available using Fried criteria [37]. The FPP is based on a pre-defined set of five criteria exploring the presence/absence of signs or symptoms (involuntary weight loss, exhaustion, slow gait speed, poor handgrip strength, and sedentary behaviour).

Current CD4 count was measured as a continuous variable: for descriptive purpose, we reported CD4 count with actual values, while it was rescaled to 100 cell/μL when included as a covariate in linear regression model. Other covariates were age and duration of HIV infection (measured continuously) and sex.

We used Generalized Estimating Equations (GEE) to assess relationships between frailty index score and current CD4 count, adjusting for age, sex and duration of HIV infection, and visualized this relationship using Lowess curves. In this model, each observation was computed as a single measurement, with no adjustment to weight multiple observation for each patient. Based on the shape of the resulting curve, we identified the strength of relationships between CD4 count and frailty in both down-sloping and up-sloping portions of the curve. Statistical significance was set at p ≤ 0.05. Data were analyzed using STATA Software package, Intercooled version 13.1 for Mac (Stata Corp ltd, Collage Station, TX, USA).

The Research Ethics Board of the University of Modena and Reggio Emilia provided approval for the Modena HIV Metabolic Clinic Cohort Study. All study participants provided written consent.

## Results

We included 2,915 participants, accounting for 10,686 total observations with median number of 4 observations per patient (IQR 2–7, range: 1–16). The observation follow-up period was 4.2 years (IQR 2.1–6.1). In this time frame 36 patients died: 11 of cancer, 4 of CVD, 5 of liver failure, 1 1 in a motor vehicle collision, 15 of unknown causes.

The mean age of the cohort at last observation was 48 ± 8.2, and 68% were men.

Table 1 summarizes demographic and clinical characteristics of the study population at baseline: median current CD4 count was 567 (418–747), current CD4/CD8 = 0.71 (IQR 0.48–1.00, data available in 2030 observations, and median duration of HIV infection was 19 (IQR 13–24) years.

**Table 1:** Demographic and clinical characteristics of the study population at baseline.

	Frequency or mean or median	% or SD or IQR
Median follow-up, years	4.2 years	2.1–6.1
Women, n (%)	869	31,8
Men, n (%)	1861	68,2
Age, mean, years	46,15	7,5
Non smoker, n (%)	693	58,6
Smoker, n (%)	489	41,4
Physical activity, n (%)	488	41,2
Sedentary life, n (%)	695	58,8
No alcohol, n (%)	699	59
Alcohol use >20 g/day, n (%)	475	40,1
Waist circumference, cm, mean	86,3	9,8
Body mass index, kg/m^2^, mean	23,4	3,6
No lipodistrophy, n (%)	563	26,4
Lipoatrophy, n (%)	696	32,6
Lipohypertrophy, n (%)	209	9,8
Mixed Form, n (%)	668	31,3
Fasting glucose, mg/dl, mean	96,2	20,2
HOMA, median	2,4	1,4–4
Triglycerides, mg/dl, median	138	96–203
Total cholesterol, mg/dl, mean	191,8	44,8
HDL cholesterol, mg/dl, mean	47,9	15,6
LDL cholesterol, mg/dl, mean	116,9	36,1
CD4 nadir, c/μL, median	196	81–297,5
Current CD4, c/μL, median	567	418–747,5
CD4/CD8 ratio, median	0.71	0.48–1.00
Cumulative exposure to NRTIs, months, median	100	48–148
Cumulative exposure to PIs, months, median (IQR)	38	7–77
Cumulative exposure to NNRTIs, months, median (IQR)	18	0–56
No hypertension, n (%)	1974	72,3
Hypertension, n (%)	756	27,7

At last visit median CD4 was 639 (IQR 466–835) and Cd4/CD8 was 0.80 (IQR 0.56–1.10; data available in 2,494 observations).

At baseline, median frailty index score was 0.31 (IQR 0.24–0.39), PFP was available in 482 patients. As classified by the frailty phenotype categories, 3.1% (15) of participants were considered to be frail, 51.9% (250) were pre-frail, and 45.0% (217) of participants were robust.

**Fig 1:**
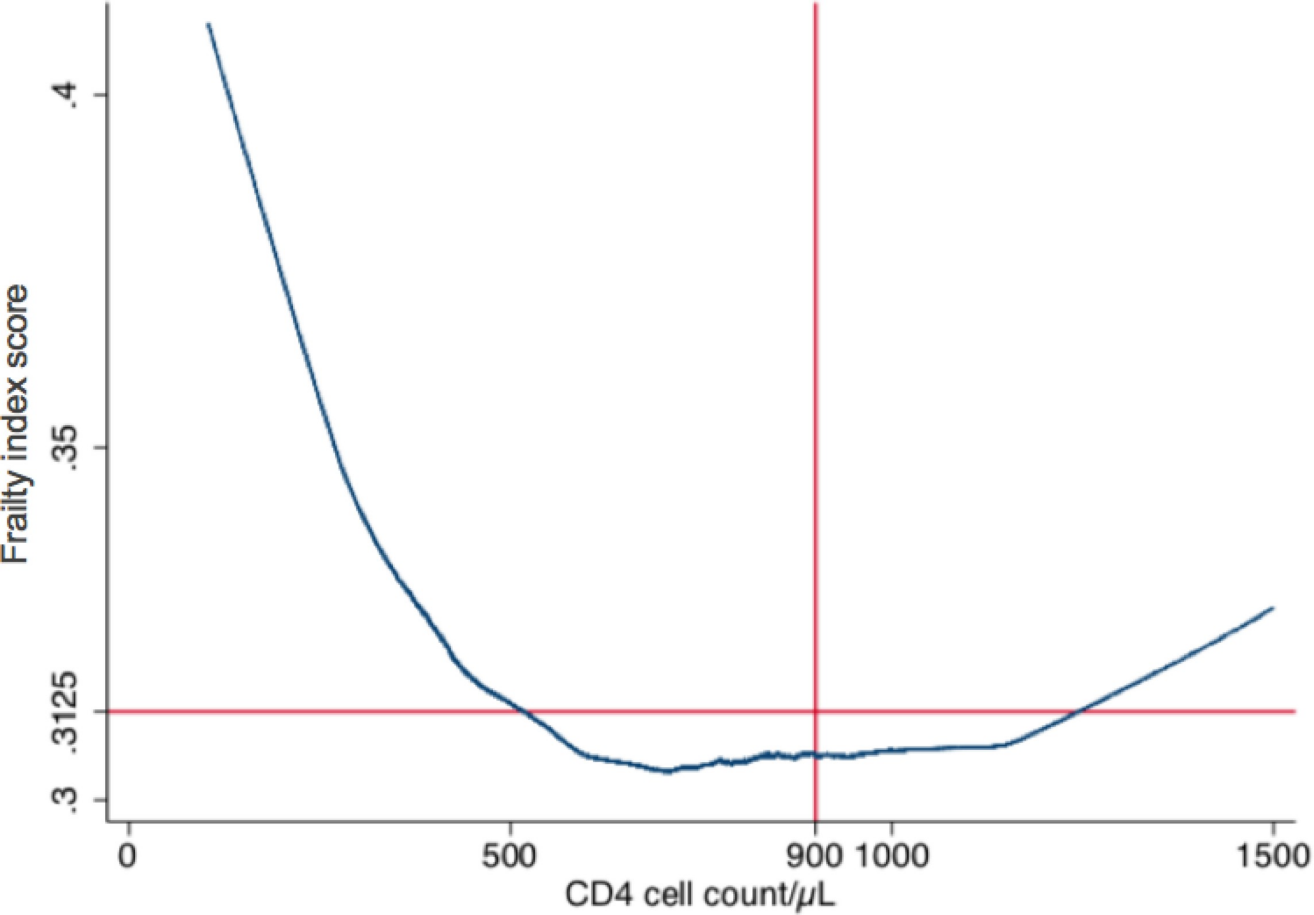
Relationship between mean frailty index and current CD4 cell count. Using a Lowess smoothing curve, frailty index exhibited a hook-shaped relationship with current CD4 count.

Using a Lowess smoothing curve, frailty index exhibited a hook-shaped relationship with current CD4 count (Fig 1). S1 Fig restricts this association at first (panel A) and last (panel B) observation per patient.

After visualization of the Lowess curves, we generated different models with different cut-offs of CD4 cell count near to the turning point of the curve. We identified a threshold of 900 CD4 cell/μL as the best turning point of ß coefficients for CD4 (Fig 1). 517 participants had current CD4 counts above 900 cells/μL at last visit.

Frailty index (per 0.01 increase) and CD4 count had an inverse relationship below 900 cells/μL (a total of 9048 observations were analysed, ß = -0.45, -0.58 to -0.32, p<0.001, per 100 cells/μL) and a borderline relationship above 900 cells/μL (1638 observations, ß = 0.23, 0.0002 to 0.46, p = 0.049, per 100 cell/μL) after adjustment for age, sex, and duration of HIV infection.

**Fig 2:**
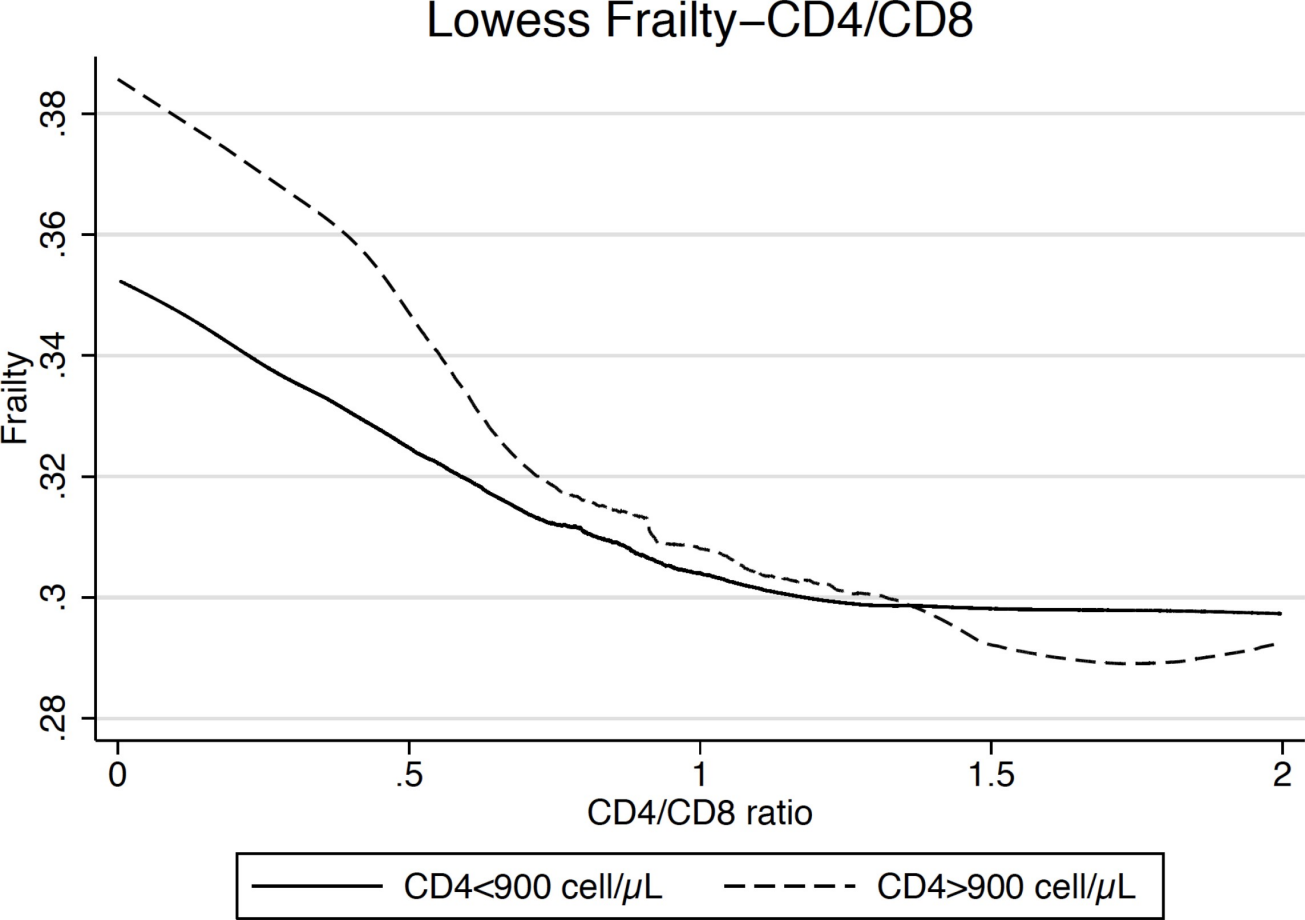
Relationship between mean frailty index and CD4/CD8 ratio. Frailty index exhibites a hook-shaped relationship with current CD4 count.

In a subset of 2756 participants accounting for a total of 8975 observations, we evaluated the association between frailty and the CD4/CD8 ratio below and above the level of 900 CD4 cells/μL. Lowess graph was drawn to depict the relationship between frailty index and CD4/CD8 ratio (Fig 2).

**Fig 3:**
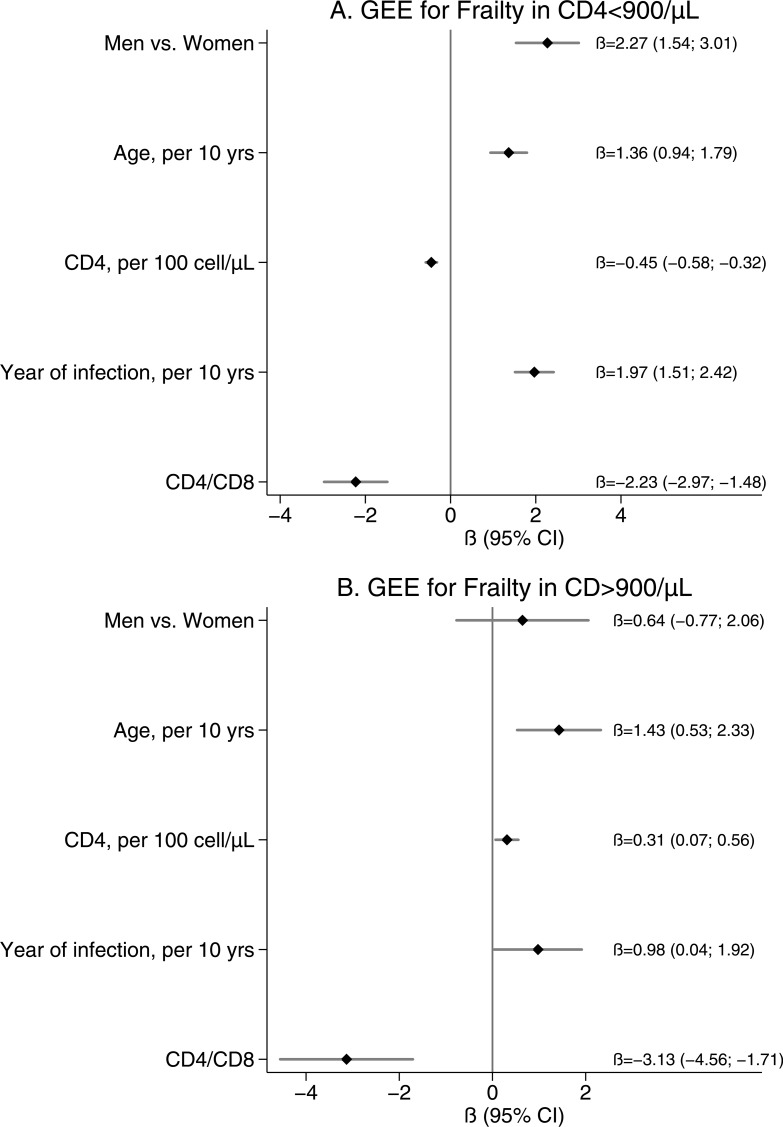
GEE linear regression estimates in subpopulation with CD4/CD8 ratio. Panel A—GEE linear regression estimates for people with CD4 cell counts less than 900/μL. Panel B—GEE linear regression estimates for those with CD4 cell counts >900/μL.

This sample was used to build multivariable GEE linear regression to explore the association between frailty index score and CD4 <900 cells (Fig 3A) and CD4 >900 cells (Fig 3B) respectively, after adjustment for age, sex, and duration of HIV infection.

In the first model (Fig 3A) frailty index score (per 0.01 increase) was inversely related with CD4 count (per any 100 cells increase) ß = -0.45 (-0.58; -0.32, p<0.001) and CD4/CD8 ß = -2.23 (-2.97; -1.48, p<0.001). It was also directly related to age ß = 1.36, male sex ß = 2.27 and duration of HIV infection (per 10-year increment) ß = 1.97.

In the second model (Fig 3B) frailty index score was only borderline related with CD4 count ß = 0.31 (0.07; 0.056) and inversely related with CD4/CD8 ß = -3.13 (-4.56; -1.71, p<0.001) and directly related to age ß = 1.43 (0.53–2.33).

## Discussion

We analysed a large sample of people aging with HIV, with a relatively low prevalence of phenotypically frail individuals.

We identified a hook-shaped relationship between severity of frailty (assessed by a frailty index of 37 health variables that describes potential age-related health problems) and current CD4 count.

In this study, we chose to describe frailty using a validated frailty index which has the advantage of being a continuous variable to be plotted with the numerical value of CD4 or CD4/CD8. We arbitrary defined frailty using a cut off value above the median in the cohort (FI>0.31). This frailty categorisation is not comparable with the physical frailty phenotype case definition, nevertheless it can be understood as the pre-frailty status depicted by Fried definition and present in 52% of our patients’ cohort. However, we have previously shown in a study comparing FI and PFP evaluated in the same population that FI had a stronger association with age, nadir CD4 count, comorbidities, falls, and disability, suggesting that this tool is more applicable in clinical settings [38].

An inverse relationship between frailty severity and CD4 count was identified below a cut-off of 900 cells/μL. We believe that this cut off is clinically significant to be used in PLWH. First, this value is universally recognised as acceptable even in HIV negative individuals. Second, this value is increasingly reached in more recently HIV-infected individuals who promptly start ART [39]. Third, it has been shown that PLWH with CD4 count between 500 and 750 cells still display increased risk of AIDS compared to PLWH with higher CD4 counts [40].

The inverse relationship between current CD4 count and frailty severity was not evident above 900 cells/μL, and a borderline-significant positive relationship was observed after adjustment for covariates. This observation requires more investigation. Our interpretation is that CD4 count above 900 cell/μl is not a prognostic marker in aging PLWH and in our model the major driver for frailty in this population are rather traditional risk factors as age and gender.

Previously published data on the relationship between the degree of frailty and CD4 count have shown varied results. Inverse relationships between current CD4 count and frailty have been identified among patients presenting for HIV care [3,6,8,9], people living with HIV in the community [10], and HIV-positive cohorts of men who have sex with men [5,24], in women [7], and in people who inject drugs [4]. Other recent studies have conversely identified no relationship between current CD4 count and frailty. Among studies of HIV outpatients [11,13], HIV-positive injection drug users [14], and men who have sex with men [12], frail and non-frail participants were similar in terms of age and current CD4 count, but differed in sex, comorbid conditions, smoking status, and markers of immune activation and inflammation.

Interestingly a marker of immune activation, namely CD4/CD8 ratio is always associated with frailty, regardless current CD4 cell count.

One previously reported study identified an inverse relationship between CD4/CD8 ratio and frailty among people with HIV [7]. A second study identified a non-significant trend towards lower CD4/CD8 ratios in people who were identified as frail compared to those who were non-frail [6].

A desirable intervention to prevent or delay the progression of frailty in HIV patients is early initiation of antiretroviral therapy, supporting recovery of the immune system. This strategy, already supported by START trial [41] should be confirmed with regards to this outcome.

Our data should be interpreted with caution, given the cross-sectional nature of the study: inferences about causality and the independent contributions of immune status and of aging to frailty cannot be assessed. More longitudinal research on the relationship between immune status and frailty is needed.

In conclusion, our findings of a dynamic relationship between current CD4 count and frailty highlight the complexity of the relationship between immune function, aging, and frailty among PLWH. We identified a cut-off of 900 cells/μL, below which further depletion of CD4 cell count is associated with increased frailty. Lower CD4/CD8 ratio, a surrogate marker of immune activation, is associated with severity of frailty both below and above this cut-off of 900 CD4 cells/μL.

## Supporting information

S1 Table Frailty index including 37 health variables. A frailty index calculates the proportion of age-related health deficits that a person has accumulated out of a selection of 37 health variables.
(DOC)

S1 Fig Association between frailty index and current CD4 cell count. Frailty index and current CD4 cell count at first (panel A) and last (panel B) observation per patient.
(DOCX)
