# Supplementary material for: The dynamic association between Frailty, CD4 and CD4/CD8 ratio in people aging with HIV
Source: PLoS One. 2019 Feb 14;14(2):e0212283. doi: 10.1371/journal.pone.0212283 (PMC6375603; doi:10.1371/journal.pone.0212283)
Supplement: S1 Table — A frailty index calculates the proportion of age-related health deficits that a person has accumulated out of a selection of 37 health variables. (DOC) [file pone.0212283.s001.doc]

| 37-item frailty index |  |
| --- | --- |
| 1 | Lipoatrophy |
| 2 | Lipohypertrophy |
| 3 | Nonalcoholic fatty liver disease |
| 4 | Menopause or male hypogonadism |
| 5 | High or low BMI |
| 6 | High waist circumference |
| 7 | High visceral adipose tissue |
| 8 | Sarcopenia or presarcopenia |
| 9 | Insulin resistance |

1. High total cholesterol
2. High low-density lipoprotein
3. Low high-density lipoprotein
4. High triglycerides
5. High homocysteine
6. Abnormal white blood cell counts
7. Anemia
8. Hepatitis C coinfection
9. Hepatitis B coinfection
10. Vitamin D insufficiency
11. Polypharmacy
12. Abnormal parathyroid hormone
13. Elevated D-dimer
14. Elevated C-reactive protein
15. Sedentary lifestyle
16. Atherosclerosis
17. Hyponatremia
18. Proteinuria or albuminuria
19. Elevated aspartate transaminase
20. Elevated alanine transaminase
21. Abnormal alkaline phosphatase
22. Elevated g-glutamyl transphosphatase
23. Low platelets
24. Abnormal potassium
25. Abnormal phosphorus
26. Abnormal thyroid-stimulating hormone
27. Elevated total bilirubin
28. Unemployment

Multicenter AIDS Cohort Study (MACS) criteria

MACS criteria

Liver/spleen ratio <1.1

If female: FSH > 30 IU/l and LH < 30 IU/l and/or absence of menstruation >1 year

If male: testosterone < 300 ng/dl

<18 or >25 kg/m^2^

If female: >88 cm

If male: >102 cm

VAT > 130 cm^2^ or VAT/TAT ratio >0.5

Fat-free mass index < 1 SD

Homeostasis Model Assessment – Insulin Resistance > 2.8

>200 mg/dl

>100 mg/dl

<40 mg/dl

>150 mg/dl

If female: >10 mmol/l

If male: >15 mmol/l

<4000 cells/ml

If female: <10 g/dl

If male: <12 g/dl

Positive

Hepatitis B antigen positive

<30 ng/ml

>5 drug classes (excluding antiretroviral therapy)

>60 pg/ml

>Sample mean (358)

>0.7 mg/l

- 3 h/week physical activity

Coronary artery calcium score > 100 or intima media thickness > 0.85 mm

<125 mmol/l

>5 mg/mmol

>31 U/l

>31 U/l

<38 or >126 U/l

>55 U/l

<150 billion/l

<3.5 or >5.3 mEq/l

<2.5 or >5.1 mg/dl

<0.27 or >4.2 mIU/l

>1 10 mg/dl

Self-report

FSH, follicle-stimulating hormone; LH, luteinizing hormone; SD, standard deviation; VAT/TAT, visceral adipose tissue/total adipose tissue.
