# Supplementary figures and images for: The dynamic association between Frailty, CD4 and CD4/CD8 ratio in people aging with HIV
Source: PLoS One. 2019 Feb 14;14(2):e0212283. doi: 10.1371/journal.pone.0212283 (PMC6375603; doi:10.1371/journal.pone.0212283)

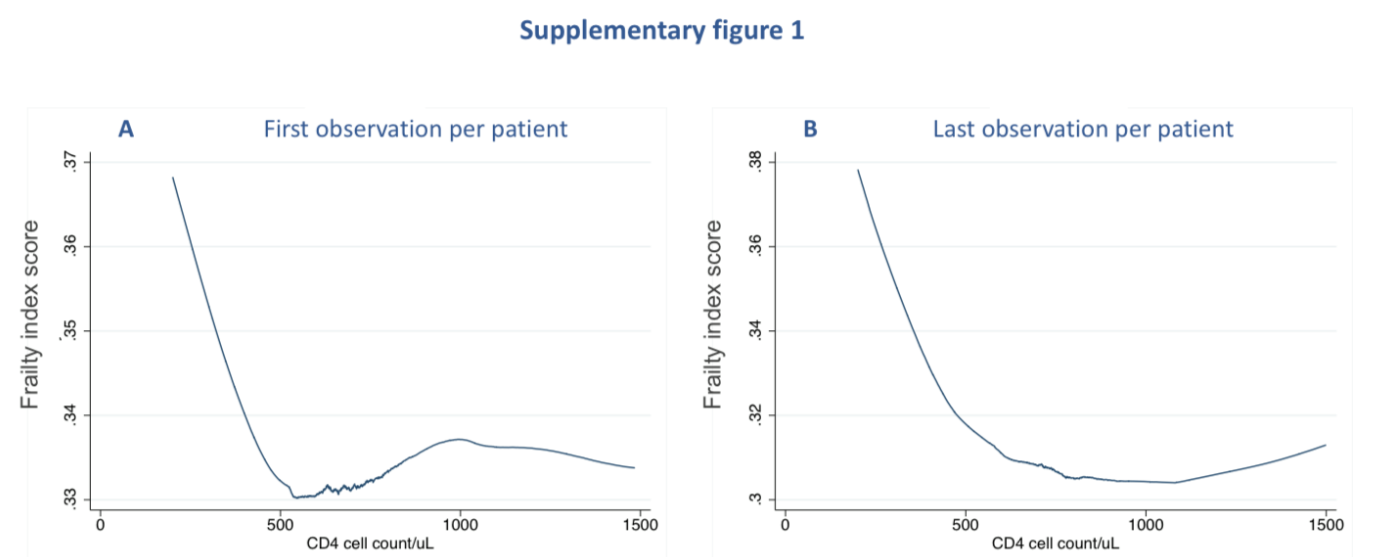

Supplement: S1 Fig — Frailty index and current CD4 cell count at first (panel A) and last (panel B) observation per patient. (DOCX) [file pone.0212283.s002.docx]
